# The Utilization of Handheld Ultrasound Devices in a Prehospital Setting

**DOI:** 10.1017/S1049023X22000644

**Published:** 2022-06

**Authors:** Kamonwon Ienghong, Lap Woon Cheung, Somsak Tiamkao, Vajarabhongsa Bhudhisawasdi, Korakot Apiratwarakul

**Affiliations:** 1. Department of Emergency Medicine, Faculty of Medicine, Khon Kaen University, Khon Kaen, Thailand; 2. Accident & Emergency Department, Princess Margaret Hospital, Kowloon, Hong Kong; 3. Emergency Medicine Unit, Li Ka Shing Faculty of Medicine, The University of Hong Kong, Pokfulam, Hong Kong; 4. Department of Medicine, Faculty of Medicine, Khon Kaen University, Khon Kaen, Thailand

**Keywords:** diagnosis, Emergency Medical Services, handheld ultrasound, prehospital emergency care, ultrasonography

## Abstract

**Introduction:**

Prehospital ultrasounds can be considered a new form of diagnostic tool when taking into account their small structure and due to the fact that nowadays, they are used in the care of emergency patients. However, at present, there is no study regarding the advantage of ultrasound usage in prehospital settings in Thailand.

**Study Objective:**

This study aims to determine the sonographic characteristics recorded by handheld ultrasounds used in prehospital care and the diagnostic accuracy of ultrasounds for prehospital patients.

**Methods:**

A cross-sectional study was conducted on prehospital patients who underwent point-of-care ultrasound (POCUS) examination on Emergency Medical Service (EMS) operations at Srinagarind Hospital, Thailand from January 2021 through December 2021. The ultrasound images, the electronic emergency department medical records, and the EMS database were recorded and reviewed by a team of emergency physicians. The quality of prehospital ultrasound examinations was assessed by comparing the diagnoses at the scene with those taken at the hospital.

**Results:**

One hundred sixty-nine prehospital patients who received POCUS examinations were examined over a one-year period. All (100.0%) of the scans were for medical cases. No ultrasound protocol was used in the prehospital care. Two hundred eight POCUS examinations were performed in this study. The most common POCUS indication was dyspnea (45.6%), followed by hypotension/shock (30.1%), and finally syncope (8.2%). The most common area where POCUS was performed was on the lung (37.0%), followed by the inferior vena cava (30.8%), and finally for cardiac cases (26.4%). This study found that 34.9% of sonographic findings could be considered abnormal. The diagnoses of prehospital patients were confirmed by using POCUS in 66 cases (39.1%) with the accuracy of prehospital diagnosis reaching a peak of 75.8%.

**Conclusion:**

This study shows POCUS examinations can be effectively used in prehospital care. The prehospital diagnosis given by physicians administering treatment who used POCUS examinations correlated with the in-hospital diagnosis.

## Introduction

Point-of-care ultrasound (POCUS) has seen a sharp increase in usage for emergency patients over the past two decades.^1–3^ Any recent studies have demonstrated the ability for POCUS to improve diagnostic accuracy,^4,5^ provide crucial information for management change, and may reduce mortality rates in critically ill and emergency patients.^6^ However, providing care to prehospital patients was extremely challenging. Emergency Medical Services (EMS) are the type of operations that necessitates quick decision making to offer emergency treatment to patients in distress.

Research shows POCUS is an effective tool when it comes to performing a quick initial evaluation, detecting aberrant pathology, and making a diagnosis in both cases of medical illness as well as for injured patients. Moreover, prehospital ultrasonography has been proved in numerous studies^7–9^ to be feasible for a wide range of operators, including flight attendant nurses, paramedics, and emergency medicine physicians.

In Thailand, over the past decade, ultrasonography education has become more accessible to emergency physicians leading to a dramatic increase in the use of ultrasound in the emergency department. This new technology also includes the handheld ultrasound device that has been introduced to university centers. Due to recent advances, ultrasound technology is now less expensive than other diagnostic imaging equipment, making it available in a wide range of health care economies; they have also become relatively portable, which makes them ideal for the unique environments of various prehospital settings where they are being used.^10^

The body of current studies regarding portable or handheld ultrasounds is limited in number. Most studies reported include the assessment of EMS personnel to perform ultrasounds,^1,11–13^ the feasibility of ultrasounds for triage in the field setting,^14^ and the utility of ultrasounds in disaster settings.^15^ However, ultrasounds used in prehospital settings in Thailand during the initial phase of diagnosis is limited in the university centers. In addition, there have been no studies on the use of POCUS for EMS in Thailand. The primary objective of this study was to describe the characteristic of POCUS used in the care of prehospital patients and to demonstrate the diagnostic accuracy of POCUS used in a prehospital setting.

## Methods

### Design and Setting

This study was carried out as a cross-sectional, observational analysis of ultrasound images obtained from 169 patients presented to the EMS unit of Srinagarind Hospital in Khon Kaen Province, Thailand for evaluation and treatment over a 12-month period from January 2021 through December 2021. This hospital is the leading medical training hospital of Khon Kaen University and advanced tertiary care institution in Northeastern Thailand, which has an average of roughly two thousand EMS operations per year.

### Data Collection

Images were obtained using a Butterfly IQ handheld ultrasound machine (Guilford, Connecticut USA). This ultrasound device permits users to obtain two-dimensional imaging, utilizing the M-mode, B-mode, and Color Doppler mode. The preset of the ultrasound device can be set as: Abdomen, Aorta, Gall Bladder, Bladder, Cardiac, FAST, Lung, Musculoskeletal, Nerve, Obstetric, Ophthalmic, Pediatric Abdomen, Pediatric Cardiac, Pediatric Lung, Small Organ, MSK-Soft Tissue, and Vascular.

Ultrasonography was performed either at the emergency site (on scene) or during patient transport by emergency medicine residents who had completed POCUS training which included didactic lectures and hands-on POCUS practice with actual patients. These sessions were monitored by POCUS experts (emergency ultrasound certified physicians) and guidance was provided when needed. Selection of cases depended on the treating physician. Exclusion criteria included unstable trauma patients, shockable cardiac arrest patients, and patients with incomplete data. Standard treatments were given during POCUS examination including airway protection, oxygen therapy, intravenous fluid, and drug administration. Patients’ provisional diagnoses were given by treating physicians after performing POCUS in an ambulance. The POCUS images and video clips were recorded in the Butterfly IQ web-based platform, which were reviewed by POCUS experts to determine the accuracy of the interpretation. The electronic emergency department medical records and the EMS database were reviewed and extracted data including patient demographic information, type of illness, patients’ provisional diagnoses at the scene, and patients’ final diagnoses by two independent emergency physicians. Following that, the duplicate data entry was completed. In the event that the data did not match, the senior emergency physician was consulted, and the correct data were obtained.

### Study Size

The sample size was calculated based on the following formula.^16^ The estimate for P was made using data from a previously published study;^7^ it was determined that a sample size of 169 would be required. Statistical analysis was performed with Khon Kaen University license (SPSS Inc.; Chicago, Illinois USA) by IBM SPSS for Windows version 27.0. Unless otherwise stated, continuous variables are reported as mean and standard deviation (SD), and categorical variables are presented as number (n) or frequency (percent).

### Ethical Considerations

Ethical approval was provided by the Khon Kaen University Ethics Committee for Human Research (HE641323). Requirement for informed consent from the patients was waived since patient confidentiality protection had been guaranteed, as patients were not identified by name but by a unique study number.

## Results

The study was performed over a period of 12 months which included 2,012 EMS operations. A total of 1,348 patients were assessed by emergency medicine residents, with 180 of them receiving POCUS examinations. In 169 cases, POCUS images and videos were recorded.

Male patients were represented as 45.6% in this study. All patients receiving POCUS examinations were medical cases (100.0%). Most patients assessed with POCUS were triaged as Level 1 and Level 2, categorized as red (42.0%), and Level 3, categorized as yellow (58.0%), according to the Emergency Severity Index (ESI), as shown in Table 1.

**Table 1.** Demographic Data of Prehospital Patients Receiving POCUS Examination (N = 169)

Category	N (%)
Sex	
Male	77 (45.6)
Mean age (SD)	57 (22)
Type of EMS Operation	
Medical Cases	169 (100)
Triaged Level	
1 and 2	71 (42)
3	98 (58)
4	0 (0)
5	0 (0)

Abbreviations: EMS, Emergency Medical Services; POCUS, Point-of-Care Ultrasound.

The prehospital emergency ultrasound was most used in patients with dyspnea (45.6%), hypotension/shock (30.1%), and syncope (8.2%). There was no ultrasound protocol used in a prehospital setting. There were 208 POCUS examinations performed in this study, which showed that the most common area where POCUS was performed was the lung (37.0%), followed by the inferior vena cava (30.8%), and finally for cardiac (26.4%). This study demonstrated that 34.9% of findings were abnormal sonographic findings (Table 2).

**Table 2.** Characteristics of POCUS Performed in EMS Patients

	N (%)	Abnormal Sonographic Findings, N (%)	
**Indication of POCUS Used (N = 169)**			
Tachypnea/Dyspnea	77 (45.6)	26 (33.7)	
		B - Lines	21
		Pleural Effusion	6
		Consolidation	2
		Pericardial Effusion	2
		Right Ventricle Dilatation	2
		Impaired Left Ventricular Contraction	6
Chest Pain	8 (4.7)	2 (25)	
		Impaired Left Ventricular Contractile Function	2
Abdominal Pain	7 (4.1)	4 (57.1)	
		Intra-Abdominal Free Fluid	2
		Abdominal Aortic Aneurysm	1
		Gall Stone	1
Syncope	14 (8.2)	6 (42.9)	
		Small Inferior Vena Cava	5
		Impaired Left Ventricular Contractile Function	1
Alteration of Conscious	2 (1.1)	0	
Hypotension/Shock	51 (30.1)	16 (31.4)	
		Small Inferior Vena Cava	12
		Impaired Left Ventricular Contractile Function	1
		Right Ventricle Dilatation	1
		Intra-Abdominal Free Fluid	2
Fever	10 (5.9)	5 (50)	
		Small Inferior Vena Cava	5
**Area of POCUS Examination (N = 208)**			
Cardiac	55 (26.4)	14 (25.5)	
Lung	77 (37)	23 (29.9)	
Inferior Vena Cava	64 (30.8)	22 (34.4)	
Abdomen (liver, gall bladder, intra-abdominal free fluid)	12 (5.8)	6 (50)	

Abbreviations: EMS, Emergency Medical Services; POCUS, Point-of-Care Ultrasound.

In patients who underwent prehospital POCUS examinations, treating physicians confirmed the prehospital diagnosis in 66 cases (39.1%). The accuracy of prehospital diagnosis was 75.8% (50/66). In 28 out of 77 (36.4%) patients who presented with tachypnea/dyspnea, the prehospital diagnosis was confirmed as pneumonia (8/12; 66.7%), heart failure (4/6; 66.7%), and volume overload (9/9; 100.0%). One patient who came with tachypnea and one patient with hypotension were confirmed to be diagnosed as having a pulmonary embolism. In two out of eight cases (25.0%) that presented with chest pain, the diagnosis of acute coronary syndrome was confirmed. Prehospital POCUS examination demonstrated intra-abdominal free fluid in four cases, which confirmed the diagnosis as ruptured ovarian cyst and spontaneous bacterial peritonitis. In six out of 14 cases (42.9%) that presented with syncope, the treating physician arrived at the diagnosis as hypovolemia (5/5; 100.0%) and cardiogenic syncope (1/1; 100.0%). In 12 out of 51 cases (42.9%) that presented with hypotension/shock, small inferior vena cava was detected and confirmed the diagnosis as hypovolemic shock (9/12; 75.0%).

## Discussion

This study highlighted the utility of POCUS examination in prehospital care. There are two main objectives: firstly, describe the characteristic of POCUS used in prehospital care, and second, determine the correlation between prehospital diagnosis given by treating physicians who used POCUS examination in patient care and in hospital diagnosis results.

This study demonstrated prehospital patients who received POCUS examinations were all medical cases, which was in contrast to previous studies.^7,17–19^ This may be due to the nature of EMS operations in trauma patients in Thailand, which are usually performed as “Scoop and Run” in which treating physicians perform Focused Assessment with Sonography for Trauma (FAST) examination in a hospital setting after the primary survey according to Advanced Trauma Life Support (ATLS) protocol.^20^ No ultrasound protocol was used in this study, which was in contrast to other studies.^7,21,22^ The main reason was the short transport time in Srinagarind EMS operations due to the area covered by EMS being not too far from the hospital. The most POCUS indication was dyspnea and shock, which was consistent with previous studies.^7,18^ This study did not include patients with cardiac arrest. This may be due to the fact that complicated interventions^23^ are usually performed to take care of these patients during the transport; hence, physicians could not have enough time to perform POCUS. However, previous studies^24–26^ demonstrated the feasibility and the utility of POCUS in out-of-hospital cardiac arrest patients, which showed the advantage of POCUS used.

This study recorded 34.9% of sonographic findings as abnormal and the accuracy of prehospital diagnosis was 75.8%. In another study,^7^ the prehospital diagnosis was confirmed in almost 90% of cases. This study has shown lower percentages of the diagnostic accuracy. The main reason may be due to the emergency medicine residents who were treating physicians at that time were pioneering the use of handheld ultrasound in prehospital care, which likely affected the sonographic skill. Another reason may be due to the limit of operation area in the ambulance, the limitation of brightness in the ambulance, and the physicians’ pressure from time constraints.

In Thailand, this study was the first prehospital POCUS study which demonstrated the correlation between the diagnostic accuracy of prehospital diagnosis and in hospital diagnosis. There is no formal POCUS training program for EMS providers.^27^ Training courses in POCUS were limited in the medical training center, especially for emergency medicine residents.^28^ This initial effort to apply POCUS in prehospital care needs to be reproduced in the community to improve the current situation. The trained emergency medicine physicians could be potential trainers in future training programs for the other levels of EMS providers.

## Limitations

The study’s limitations were: (1) data collection from a single EMS, single level of EMS personnel, which may have a different perspective on the studied population than other organizations – as a result, data should be gathered from a variety of research organizations; (2) this study did not demonstrate the quality of image acquisition acquired from other handheld ultrasound devices; (3) this study did not determine the effect of POCUS examination to the time of EMS operation; and (4) this study did not identify the association of POCUS examination among the change of patient management and the patient outcome.

## Conclusions

Handheld POCUS can potentially be an additional diagnostic tool in the prehospital setting. The POCUS examinations revealed significant pathology that aided treating physicians in giving correct diagnoses. Future studies are needed to determine what role POCUS may play in prehospital care.
