# The Utilization of Handheld Ultrasound Devices in a Prehospital Settings – CORRIGENDUM

**DOI:** 10.1017/S1049023X25000044

**Published:** 2024-12

**Authors:** Kamonwon Ienghong, Lap Woon Cheung, Somsak Tiamkao, Vajarabhongsa Bhudhisawasdi, Korakot Apiratwarakul

The methods section in both the abstract and the main text of the document currently read the study was conducted “from January 2021 through December 2021;” the authors would like to correct this to read “from January 2020 through December 2020.”
